# Implications of high rates of sexual recruitment in driving rapid reef recovery in Mo’orea, French Polynesia

**DOI:** 10.1038/s41598-018-34686-z

**Published:** 2018-11-09

**Authors:** Peter J. Edmunds

**Affiliations:** Department of Biology, California State University, 18111 Nordhoff Street, Northridge, CA 91330-8303 USA

## Abstract

Coral abundance continues to decline on tropical reefs around the world, and this trend suggests that coral reefs may not persist beyond the current century. In contrast, this study describes the near-complete mortality of corals on the outer reef (10 m and 17 m depth) of the north shore of Mo’orea, French Polynesia, from 2005 to 2010, followed by unprecedented recovery from 2011 to 2017. Intense corallivory and a cyclone drove coral cover from 33–48% to <3% by 2010, but over the following seven years, recovery occurred through rapid population growth (up to 12% cover y^−1^) to 25–74% cover by 2017. The thirteen-year, U-shape trajectory of coral cover over time created by the loss and replacement of millions of corals through sexual reproduction underscores the potential for beneficial genetic responses to environmental conditions for at least one genus, *Pocillopora*. The high ecological resilience of this coral community appears to have been enhanced by variation among genera in the susceptibility to declining cover, and the capacity for population growth (i.e., response diversity). These results suggest that the outer coral communities of Mo’orea may be poised for genetic changes that could affect their capacity to persistence.

## Introduction

The Anthropocene has brought intense disturbance to the biosphere^1^, with the most profound consequences in the ocean attributed to global warming^2^ and seawater acidification^3^. These effects have created a fingerprint of human impacts on every ecosystem, in most cases involving large changes in population size of multiple species^4^. Declines in population size attract attention when they threaten to cause local extirpation, particularly when this involves organisms with ecological or economic importance^5,6^, and such cases motivate interest in the possibilities of population recovery. In the last decade, there has been a growing consideration of whether recovery can support “rescue” from extinction through the influx of new individuals into a sink population (demographic rescue, DR), the infusion of genotypes into a small population to avoid inbreeding depression (genetic rescue, GR), or rapid adaptation through recruitment into a declining population followed by natural selection (evolutionary rescue, ER)^7–9^.

On tropical reefs, the large declines in size of populations of many corals that have occurred since the1960’s suggest that some species already may be ecologically extinct^10^, while others face high risks of extinction^11^. These trends define the modern coral reef crisis^12,13^, which refers to the global reduction in the number of reefs functionally dominated by reef-building corals. The magnitude of these changes has focused interest on reef recovery following disturbances^14^, but faced with the reality that this outcome is unlikely^15,16^, novel mechanisms are being addressed to facilitate a return to a coral-dominated state^17^. Central to these efforts is the notion that devastated coral populations no longer can recover between disturbances^16,18^, and while this is consistent with many long-term studies^19–21^, in a few locations coral communities have recovered^14,22–24^. A review of cases of reef recovery^23^ published from 1972 to 2009 found 48 cases in which the rate of increase in coral cover following major disturbances was 3.6% y^−1^, with 95% of the values between 2.9 and 4.4% y^−1^. Examples of recovery of coral communities would be particularly important if they serve to rescue populations from the risks of extinction^9^. Further, if recovery is associated with variation among functionally similar taxa in their response to environmental changes, it would underscore the potential of biological diversity to promote ecological resilience through response diversity^25^. Opportunities to explore rescue effects and response diversity are provided by the outer reefs of Mo’orea, French Polynesia, which have undergone a remarkable cycle of destruction and recovery in less than a decade^26–28^.

The present study focuses on the coral community (scleractinians and *Millepora*) on the north shore of Mo’orea, which has been intensively studied by the Mo’orea Coral Reef LTER at two sites and two depths since 2005. Portions of the data from these studies have revealed a cycle of coral mortality (from 2005 to 2010) and recovery (from 2010 to 2016)^27,29^, which is the third such cycle since the late 1970’s^28,30^ with the most recent recovery mediated by density-dependent recruitment of *Pocillopora* spp.^26,31^, and probably also for *Acropora* spp.^26^. Despite very large efforts to study the long-term ecology of the coral reefs around Mo’orea^27,28^, the most striking features of the most recent cycle of death and recovery have not been fully addressed: the abruptness of coral mortality, the extreme rapidity and relative completeness of the recovery of the coral community^26,28^ and the extent to which coral taxa responded differently to common events. Together, these features have created a striking U-shape trajectory of coral cover over 13 years^26–28^. While this trajectory is one of several features defining ER and DR, alone it cannot demonstrate that either type of rescue is underway^7,9,32^. In ER, a population facing an elevated risk of extinction through reductions in size recovers through high recruitment, gene flow, and genetic adaptation, with adaptive evolution serving as the mechanism of rescue^9^. In DR, a population facing an elevated risk of extinction through reductions in size persists through the addition of immigrants to a sink population, with immigration serving as the mechanism of rescue. In GR, a numerically small population benefits from the influx of genetic variation through immigrants, followed by hybridization that alleviates the risks of inbreeding. While it was beyond the scope of the present study to distinguish among these mechanisms, indirect evidence (described in the discussion) from Mo’orea suggests GR is unlikely, and while both ER and DR might be possible, ER is more likely than DR. If ER underlies the pattern of recent changes affecting the coral communities of Mo’orea^26–28^, then not only might this location provide an example of a coral reef oasis^33^, but it also could be a site at which corals have the potential to undergo rapid genetic adaptation to changing environmental conditions which function as a selective forces (e.g., globally rising seawater temperature^34^).

While the trends affecting the outer reefs of Mo’orea can be summarized using coral cover, as is common in coral reef ecology^16^, in this location, the taxonomic identity of corals has mediated recent changes in coral cover. For example, *Pocillopora* spp. recovered more rapidly that *Acropora* spp.^26,28^, and *Millepora* sp. escaped predation by the sea star *Acanthaster planci*^35^ (hereafter COTS). Against this backdrop, the objectives of the present study were two-fold: (1) to describe the dynamics of coral communities on the outer reefs of Mo’orea with genus resolution, and evaluate the extent to which their recent response to disturbances is indicative of ER, and (2) to explore the role of response diversity among coral genera in community dynamics.

## Results

### Overview of sampling

This analysis extended over 13 years, in each of which the reef was sampled for community structure and coral recruitment. About 40 independent photoquadrats were recorded annually at each site and depth to provide a ~2,080 images, within which, ~416,000 decisions were made concerning types of organisms covering the benthos. Coral recruitment was recorded over each sampling interval using ~15 tiles at each site and depth, with tiles changed twice yearly. Recruit sampling supported calculations of annual recruitment at 10-m depth from January 2008, and at 17-m depth from January 2009. About 570 settlement tiles we screened to generate annual rates of coral recruitment.

### Overall community structure

**Figure 1:**
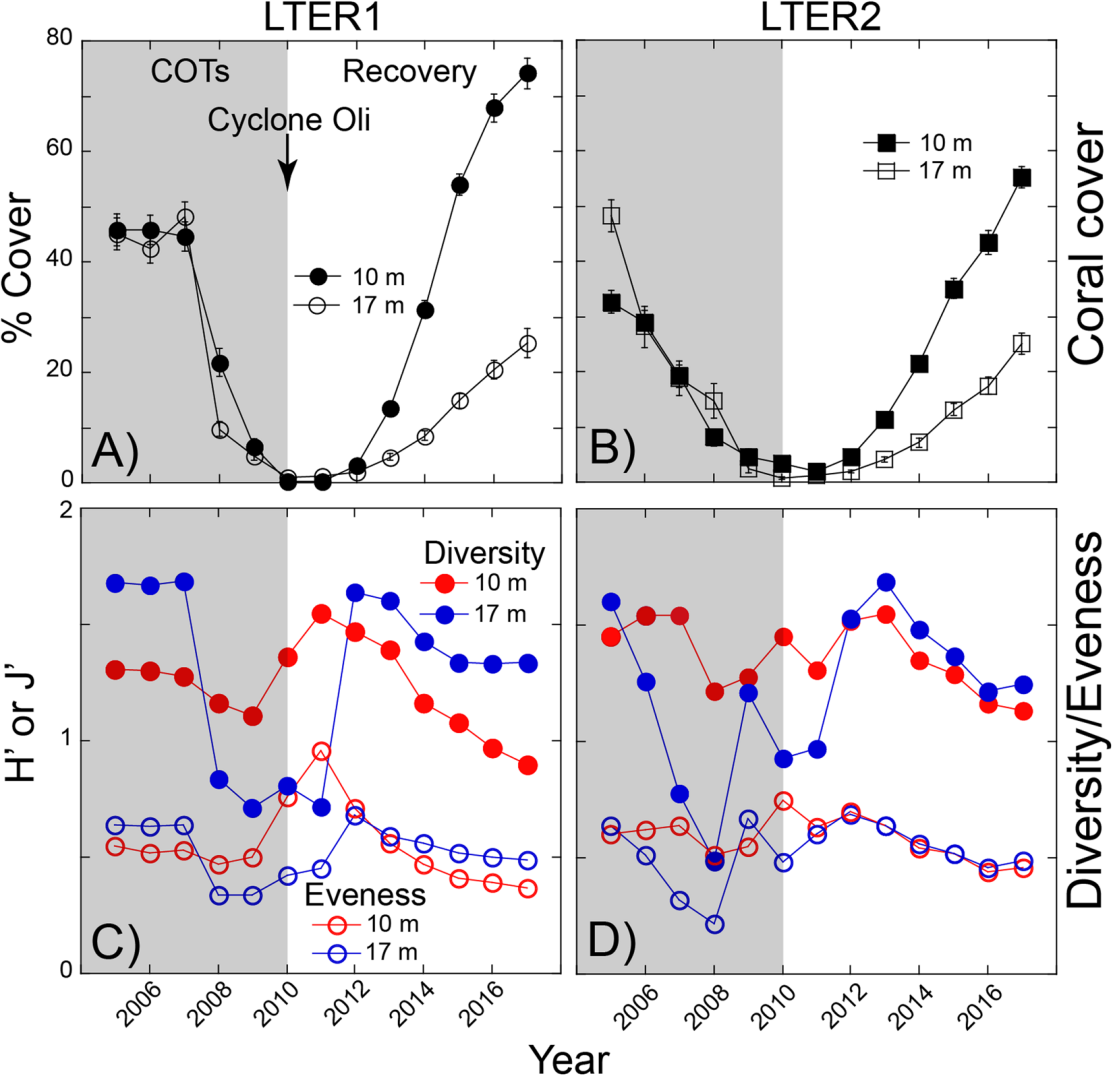
Community structure at 10-m and 17-m depth at LTER 1 and LTER 2 from 2005 to 2017. (**A**,**B**) Coral cover (scleractinians and *Millepora*), mean ± SE (n = 36–40 y^−1^), and (**C**,**D**) Shannon-Wiener diversity (H′, blue) and Pielou’s Evenness (J′, red). Shaded blocks show the outbreak of the crown-of-thorns (COTs) sea star, followed by Cyclone Oli.

The coral community changed rapidly at both sites and depths, with coral cover following a U-shape trajectory. At LTER1 in 2005, mean cover was 46% at 10-m depth, and 45% at 17-m depth, but declined to <1% in 2010 due to corallivory by COTS, followed by Cyclone Oli in February 2010. Recovery began with sexual recruitment, and within seven years, coral cover reached 74% at 10-m, and 25% at 17-m depth (Fig. 1). The coral community at LTER2 followed similar trends, although COTS appeared earlier, and cover declined from 33% (10-m depth) and 48% (17-m depth) to ≤3% in 2010. Thereafter, it increased to 55% at 10-m depth, and 25% at 17-m depth by 2017 (Fig. 1). Coral cover changed over time in a pattern that differed between sites and depths (F_12,1812_ = 8.441, P < 0.001), and differences between sites varied between depths (F_1,152_ = 5.711, P = 0.018).

At LTER 1, coral generic diversity (Fig. 1) in 2005 was higher at 17-m (H′ = 1.7) than 10-m (H′ = 1.3) depth, at 17-m depth it declined in 2009, before rising in 2012, then declining to 1.3 in 2017. Changes in H′ were less acute at 10-m depth, and were attenuated at LTER2. At both depths, H′ declined after 2013, reaching 1.1 and 1.3 at 10-m and 17-m depth, respectively. These changes reflect the reduction of H′ as COTS consumed corals, the greater speed with which H′ increased after 2010 relative to cover, and the depression of H′ after 2012 as *Pocillopora* dominated the community. Evenness (J′, Fig. 1) showed similar trends at both sites and depths, declining as taxa were consumed by COTS, rising as fewer taxa became more equally represented, then declining with more heterogeneous taxonomic representation; J′ was lower in 2017 than 2005.

### Temporal trends by taxon

**Figure 2:**
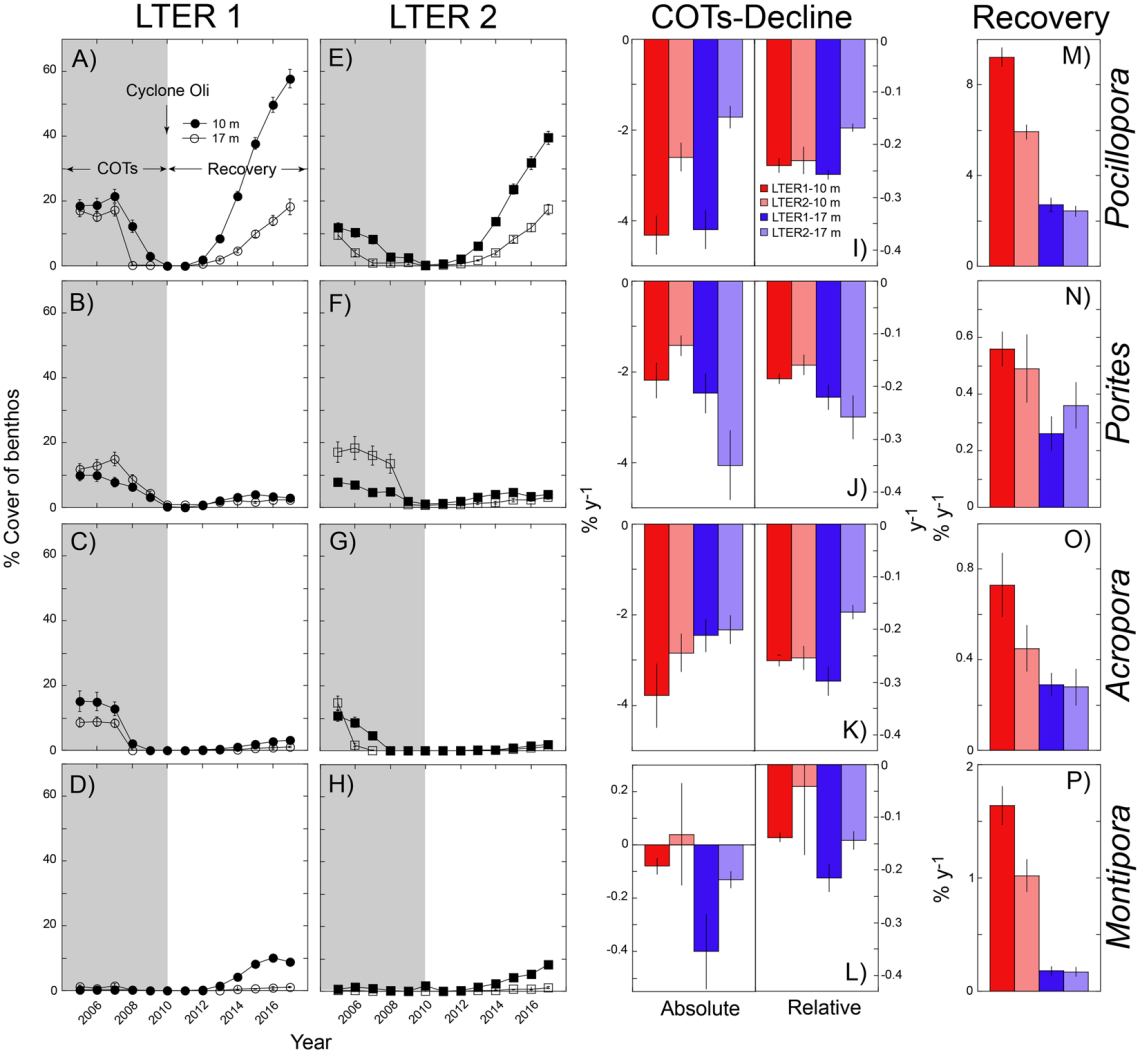
Changes in percentage cover of the benthos for the four most common coral genera at LTER1 and 2, and 10-m and 17-m depth, from 2005 to 2017. (**A–H**) Mean coral cover (±SE) by genus (rows) and sites (columns) for both depths (n = 36–40 y^−1^). Shading shows outbreak of COTs, followed by Cyclone Oli, and a period of recovery. (**I–P**) Change in coral cover by taxon (rows), depth (10 m, red; 17 m, blue), and site (LTER1, dark shading; LTER2, light shading) for COTs decline then recovery. For COTs decline, rates are in absolute (% y^−1^) and relative units (y^−1^). Slopes are shown as mean ± SE, with sample sizes of 35–40 (*Pocillopora*), 36–40 (*Porites*), 21–38 (*Acropora*), and 12–40 (*Montipora*).

Cover of the common genera changed in ways that were similar to overall cover, with a U-shape trajectory for *Pocillopora*, which accounted for a median of 42% of coral cover across the study (inter-quartile range = 30–65%) (Fig. 2A–H). The cover of *Pocillopora*, *Porites*, *Acropora*, and *Montipora* changed over time in a pattern that differed among sites and depths (i.e., the three way interactions was significant for these genera, P ≤ 0.007, Fig. 2, Table 1): *Pocillopora* varied between sites, differentially by depth; *Acropora* differed between sites and depths; and *Montipora* differed between depths (P ≤ 0.037, Table 1).

**Table 1:** Results of repeated measures (RM) ANOVA comparing percentage cover (arcsine transformed) of the four most common genera of corals among sites and depths using photoquadrats as independent replicates (Fig. 2).

Taxon	Source	MS	df	F	P
*Pocillopora*	Between				
	Site	0.827	1	15.444	<0.001
	Depth	2.261	1	42.218	<0.001
	Site × Depth	0.236	1	4.406	0.037
	Error	0.054	151		
	Within				
	Time	0.432	12	30.532	<0.001
	Time × Site	0.116	12	8.167	<0.001
	Time × Depth	0286	12	20.210	<0.001
	Time × Depth × Site	0.139	12	9.844	<0.001
	Error	0.014	1,812		
*Porites*	Between				
	Site	0.001	1	0.012	0.913
	Depth	0.021	1	0.239	0.626
	Site × Depth	0.002	1	0.024	0.878
	Error	0.088	151		
	Within				
	Time	0.035	12	2.778	0.001
	Time × Site	0.027	12	2.179	0.011
	Time × Depth	0.024	12	1.867	0.034
	Time × Depth × Site	0.029	12	2.281	0.007
	Error	0.013	1,812		
*Acropora*	Between				
	Site	0.179	1	6.054	0.015
	Depth	0.220	1	7.456	0.007
	Site × Depth	0.053	1	1.803	0.181
	Error	0.030	151		
	Within				
	Time	0.050	12	5.711	<0.001
	Time × Site	0.025	12	2.867	0.001
	Time × Depth	0.030	12	3.425	<0.001
	Time × Depth × Site	0.049	12	5.572	<0.001
	Error	0.009	1,812		
*Montipora*	Between				
	Site	0.002	1	0.276	0.600
	Depth	0.230	1	26.427	<0.001
	Site × Depth	<0.001	1	0.002	0.969
	Error	0.009	151		
	Within				
	Time	0.137	12	35.012	<0.001
	Time × Site	0.039	12	9.916	<0.001
	Time × Depth	0.094	12	24.059	<0.001
	Time × Depth × Site	0.031	12	7.986	<0.001
	Error	0.004	1,812		

**Figure 3:**
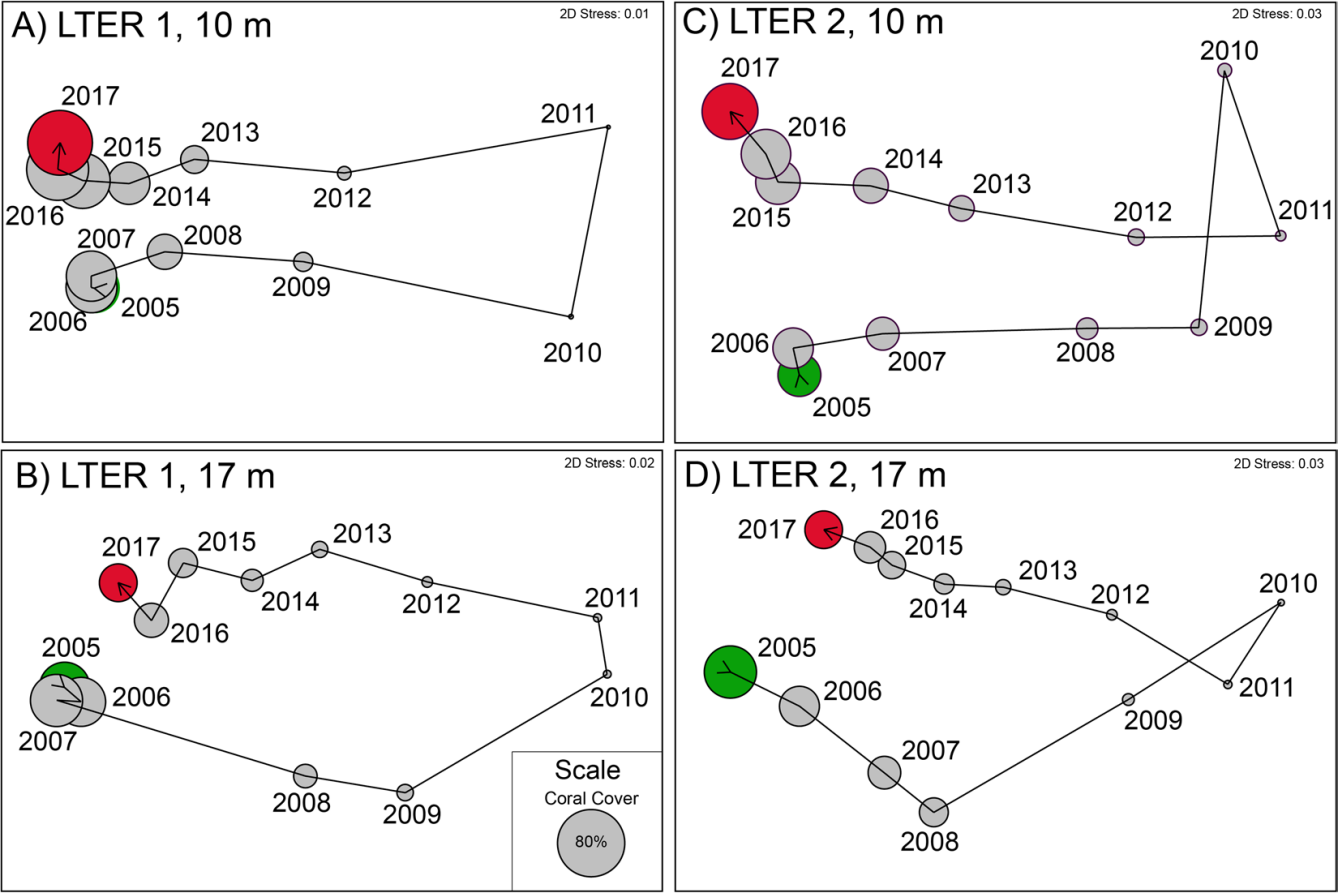
nMDS ordination of coral communities at LTER 1 (**A**,**B**) and LTER 2 (**C**,**D**) at 10-m (**A**,**C**) and 17-m **(B**,**D**) depth. Green = first sampling year, red = last sampling year, circles scaled to overall coral cover (scleractinians + *Millepora*, common scale show in (**B**)), and vectors link sequential years.

Two-dimensional nMDS ordination (Fig. 3) showed that the communities quickly diverged from their condition in 2005 as corals were consumed by COTS and dislodged by Cyclone Oli. Following these disturbances, coral communities in 2017 at both sites and depths become relatively similar to the communities in 2005 (i.e., recovery occurred), although community reassembly relatively was more complete at LTER1 versus LTER 2, and at 10-m versus 17-m depth. At 10-m depth, community structure in 2016 and 2017 started to diverge from the 2005 condition as, for example, *Montipora* occupied more space in 2016–2017 than it did in 2005 (Fig. 2D,H).

The rates at which coral cover changed (Fig. 2), underscored the extent to which corals responded differentially by taxon, site, and depth to mortality (i.e., they were differentially consumed by COTS), and then recovery. Rates of change were calculated by quadrat, and although the study was designed to sample 38–40 quadrats by depth and site every year, a few quadrats were omitted annually due to errors in field sampling. Declines in cover, therefore, were calculated with 5–6 years of data, and recovery was calculated with 7–8 years of data. Sample sizes sometimes were further reduced for each genus, either because all genera were not found in all quadrats or, in the case of declines standardized to initial cover, select genera were was not found in 2005. Of the slopes of changing cover, 45–94% (N = 140–155) were significant for *Pocillopora*, 30–38% (N = 136–152) were significant for *Porites*, 36–43% (N = 94–140) were significant for *Acropora*, and 2–28% (N = 35–144) were significant for *Montipora*.

For the period of declining population size, changes in absolute cover ranged from −1.77% y^−1^ to −4.3% y^−1^ for *Pocillopora*, −1.42% y^−1^ to −4.06% y^−1^ for *Porites*, −2.33% y^−1^ to −3.79% y^−1^ for *Acropora*, and 0.04% y^−1^ to −0.43% y^−1^ for *Montipora* (Fig. 2I–L); slopes differed between sites for *Pocillopora* (F_1,145_ = 33.128, P < 0.001), but no other contrasts for this, or any other genus were significant (P > 0.050). Changes in relative cover ranged from −0.17% y^−1^ to −0.26% y^−1^ for *Pocillopora*, −0.16% y^−1^ to −0.26% y^−1^ for *Porites*, −0.17% y^−1^ to −0.30% y^−1^ for *Acropora*, and 0.04% y^−1^ to −0.22% y^−1^ for *Montipora* (Fig. 2I–L); slopes differed between sites and depths for *Pocillopora* (F_1,129_ = 24.527, P < 0.001), and *Acropora* (F_1,119_ = 13.732, P < 0.001), but no other contrasts for these, or any other genus were significant (P > 0.050).

For the period of increasing population size, changes in absolute cover ranged from 2.43% y^−1^ to 9.23% y^−1^ for *Pocillopora*, 0.26% y^−1^ to 0.56% y^−1^ for *Porites*, 0.28% y^−1^ to 0.73% y^−1^ for *Acropora*, and 0.17% y^−1^ to 1.55% y^−1^ for *Montipora* (Fig. 2M–P); slopes differed between sites and depths for *Pocillopora* (F_1,150_ = 28.805, P < 0.001), and between depths for *Porites* (F_1,145_ = 5.664, P = 0.019), *Acropora* (F_1,90_ = 8.802, P = 0.004), and *Montipora* (F_1,83_ = 104.603, P < 0.001). Three way PERMANOVAs revealed taxon × site and taxon × depth interactions for absolute declines (Pseudo-F_3,514_ ≥ 4.39, P_perm_ = 0.002), a taxon × depth interaction for relative declines (Pseudo-F_3,514_ = 3.67, P_perm_ = 0.021), and a taxon × depth × site interaction for absolute increases (Pseudo-F_3,526_ = 2.45, P_perm_ = 0.037).

### Recruitment

**Figure 4:**
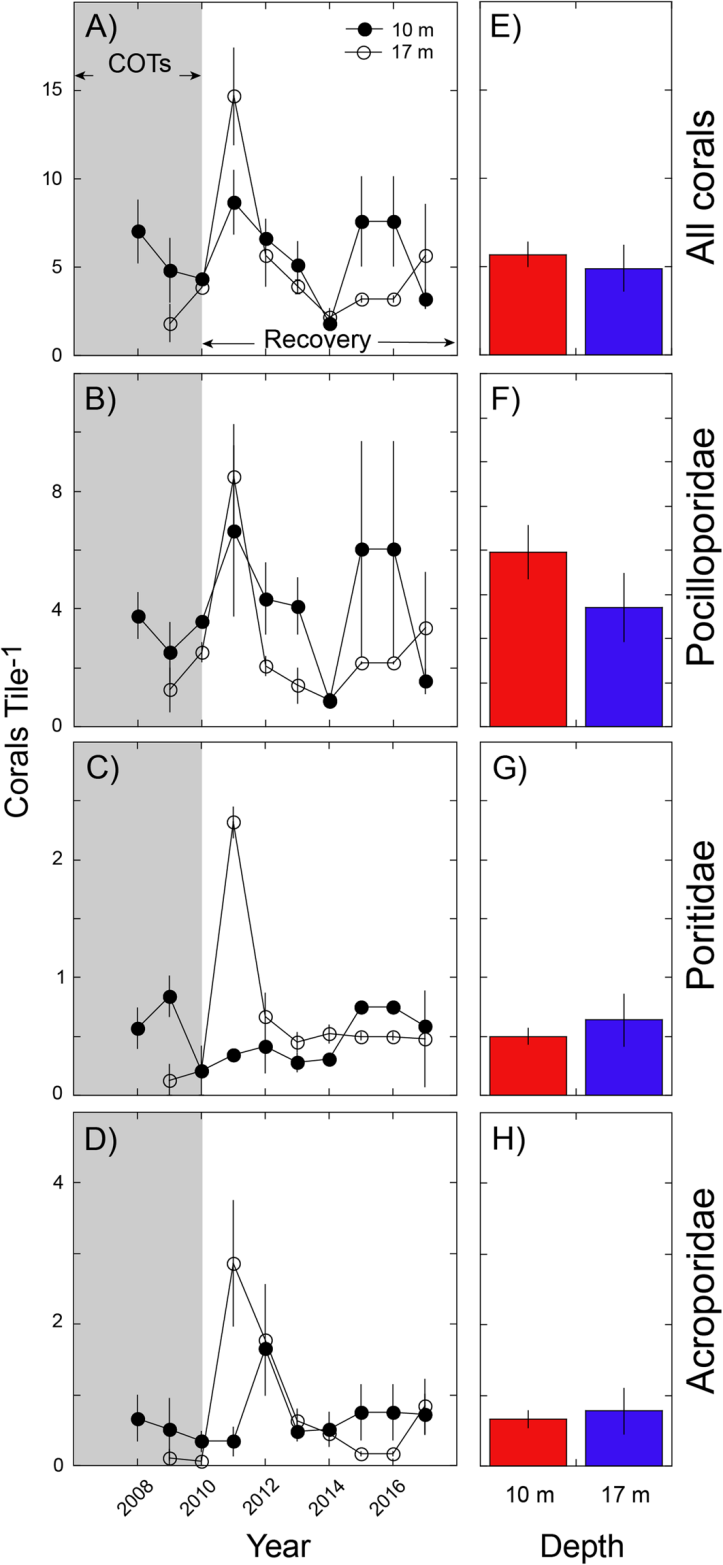
Coral recruitment by taxon (panel rows) to settlement tiles at 10-m and 17-m depth at LTER1 and 2; scales differ among taxa. (**A–D**) Mean annual recruitment (±SE, n = 2 sites) by depth and time, (**E–H**) Mean annual recruitment by depth (±SE, n = 10 [10 m] or 9 [17 m]).

Corals recruited with higher (i.e., 1.1 to 61.4-fold greater) densities from January/February to August/September than from August/September to January/February, and the majority (>80%) was found on the lower surfaces of tiles. During any one deployment, some tiles at each site and depth had no recruits, while others as many as 44 recruits, and mean densities ranged between 0.16 recruits tile^−1^ and 8.59 recruits tile^−1^. When recruits were summed within year and averaged across sites, mean densities (pooled among taxa) at 10-m depth ranged from 1.83 ± 0.30 recruits tile^−1^ to 8.67 ± 1.81 recruits tile^−1^, and at 17-m depth, from 1.83 ± 1.04 recruits tile^−1^ to 14.68 ± 2.76 recruits tile^−1^ (± SE, N = 2) (Fig. 4A–D). The density of recruits differed among years at both depths, generally with higher densities in the recovery (2010–2017) versus the decline (2008–2010) phase, higher densities 10-m versus 17-m depth during the decline phase, and a mixed pattern between depths during the recovery phase. Similar variation was displayed by three families of recruits, although pocilloporids at 10-m depth were 2–20-fold more abundant than acroporids, and 3–30-fold more abundant than poritids; at 17-m depth, the discrepancies were 1–41-fold and 2–12-fold, respectively. Over the study, densities of all recruits, as well as for the three families, were equally abundant at both depths (P ≥ 0.216) (Fig. 4E–H).

## Discussion

### Overview

Along with several other recent publications^26–28^, the present study underscore the remarkable sequence of events that have affected the outer reefs of the north shore of Mo’orea since 2005. The most unusual aspect of these events is the very high rate of return of coral cover, with the rate recorded at one site (12% y^−1^) probably one of the highest rates of recovery of a coral reef recorded to date^23^. However, there is more ecological significance to this episode of reef recovery that its rapidity alone. First, the U-shape trajectory of coral cover against time is consistent with two forms of the “rescue effect” (*sensu*^36^) – ER and DR – which have implications for the future dynamics of this coral community. Given the large size of the coral populations in 2005 before the onset of COTs and the 2010 cyclone, and the dominance of spawning and external fertilization among most of the corals in Mo’orea^26^, it is unlikely that these corals formed small and inbred populations that are integral to the function of GR^9^. While it was beyond the scope of the present analysis to distinguish between ER and DR, notably by testing for rising frequencies of adaptive alleles and phenotypes^8,9^, indirect evidence suggests that ER is more likely than DR. Second, evidence of response diversity (*sensu*^25^) among coral taxa, including variation in the extent to which the trajectories of cover are U-shaped, indicates that the strength of the putative rescue effect differs among coral taxa. Therefore, the recovery of the coral community on the outer reefs of Mo’orea may include an elevated capacity to respond to future conditions as a result of ER, driven in part, by response diversity among coral genera that promotes community resilience^37^.

While the aforementioned trends for Mo’orea differ from those on many other reefs^21,38–40^, this is the third cycle of reef recovery to occur in this location since the late 1970’s^28,30^, and globally, similar trends have been reported in a few other locations^14,22–24^. In Mo’orea, coral cover on the outer reef in 1979 was reduced from ~45% to ~12% in 1982 through corallivory by COTS, but by 1991 it had returned to 51% (at 10–12-m depth); in 1991, it was impacted by bleaching and a cyclone that reduced cover to 23% in 1993, but by 2004 it had returned to ~50%^30^. In the most recent event, recovery in places was more dramatic than previously recorded, and attenuated counter-clockwise around the island^27^. Elsewhere, examples of reef recovery in recent decades come mostly from the Indo-Pacific (with a few from the Caribbean^41,42^), and include Scott Reef on the west coast of Australia over 1995–2011^22^, 12 of 21 reefs in the Seychelles over 1994–2011^14^, Panamanian reefs following the El Niños of 1982–83 and 1997–98^43^, and reefs near Singapore over 1986–2012^24^. These examples differ from Mo’orea, however, because they developed over different scales of time or coral cover (i.e., the rate of return of coral was lower than in Mo’orea), occurred in a different habitat (e.g., a fringing reefs with turbid water and anthropogenic disturbances in Singapore), or were supported by mechanism inconsistent with genetic variation (e.g., asexual proliferation on Scott Reef and in Panama).

The present study does not focus on the recovery of Mo’orean reefs per se, as earlier stages of this trend have been reported elsewhere^26–29^. Rather, it augments interpretation of this event by describing variation in coral cover by genus and depth, it includes new information on coral recruitment over time and depths, and uses trends in these data to support the hypothesis that ER and response diversity may be enhancing community resilience. Indirect evidence is consistent with ER (as opposed to DR^7–9^) on the outer reefs of Mo’orea, including: (1) the very large size of coral populations prior to the onset of the most recent decline (i.e., they are unlikely to be sink populations as required for DR^9^), (2) the rapidity of the population decline, (3) highly fecund, sexual reproduction of multiple coral taxa, as well as larval connectivity among nearby islands, which together greatly amplify the supply of diverse coral genotypes to the reefs of Mo’orea, and (4) signs of enhanced resilience of the coral community to at least one contemporary driver of changing coral community structure, elevated thermal stress^44,45^. Together, these lines of evidence meet the three requirements that increase the likelihood of ER: large initial populations, high initial standing genetic variation, and gradually changing environmental conditions^9^. The role of response diversity in mediating spatio-temporal variation in coral cover is revealed by the extent to which this dependent variable is affected by interactions among taxon, time, site, and depths.

### Evolutionary rescue in Mo’orea?

Populations subject to large reductions in abundance are prone to extinction through random processes when densities fall below a stochastic threshold^7,9^. There are three means of “rescue” to alleviate this risk^9^, one of which is ER, which leads to an increased frequency of resistant phenotypes, thus favoring avoidance of extinction. Despite substantial research on these mechanisms^8,9,32^, few examples of ER have been reported for natural systems, in part, because demographic evidence of U-shaped trajectories is missing^9,32^, and in part because evidence of change frequencies of alleles and phenotype is lacking. To what extent then, are the recent changes in the coral communities of Mo’orea consistent with the possibility that ER could have a role in promoting persistent of coral communities in this location?

The outer reef of Mo’orea provides clear examples of U-shaped population trajectories, with only a few years below a putative stochastic threshold of ~3% cover. Pocilloporids were strong drivers of these trajectories, with lesser roles played by massive *Porites*, *Acropora*, and 16 other taxa (15 genera and Fungiidae) of scleractinians, and *Millepora*. Although pocilloporids constituted 31–78% of the coral cover from 2012–2017, the recovery of the coral community was taxonomically broad as revealed by the rebound of coral diversity and richness, and the progression of community similarity among years from near-identical (2005 vs 2006), to strikingly different (e.g., 2005 vs 2010), and back to relatively similar (2005 vs 2017). Within the Pocilloporidae, recovery involved at least five species and two un-named haplotypes^46^, and was driven by inverse density-dependence recruitment of sexual larvae^26,31^; positive density-associated recruitment delayed recovery of acroporids^26^. High coral cover on the outer reefs of Mo’orea (i.e., >25% as in 2005 and 2017) is produced by ca. 1.8 × 10^8^ pocilloporid colonies along the north shore^47^ and, therefore, the recent community hiatus involved a massive reduction of a very large population over a substantial area (i.e., ~50 km^2^). It is impossible to know whether this event reduced population sizes to levels that elevated risks of local extinction from which rescue was required. Nevertheless, the plethora of global threats to coral survival^5,13,48^, together with declines in abundance of corals in the back- and fringing- reefs of Mo’orea that were concurrent with the death of corals on the outer reef, suggest local extinction for multiple coral species in Mo’orea was one possible outcome of the 2005–2010 population collapse.

The source of larvae promoting coral recovery in Mo’orea remains equivocal. For pocilloporids, colonies in the back reef that survived COTS and Cyclone Oli could have seeded the outer reef with larvae^47^, yet genetic analyses reveal that outer reef pocilloporids sometimes are connected with corals on Tahiti^49^, but at other times are not^46^. The westward/southwestward flow of seawater from Tahiti^50^, where there also was a recent COTS outbreak and damage from Cyclone Oli, and genetic connectivity between Tahiti and Mo’orea for a variety of taxa^51,52^, increases the likelihood that coral recruitment in Mo’orea is supplied, at times, by larvae from other islands. While the environmental regimes in these nearby islands are unlikely to be profoundly different from Mo’orea and, therefore, unlikely to favor-locally adapted coral genotypes, such larval connectivity would ensure that coral recruits in Mo’orea potentially are drawn from a very large gene pool. This inference provides a mechanism by which ER for pocilloporids, and perhaps other corals, could be supported, although evidence that it has occurred – for example, through an increased frequency of resistant phenotypes – has not yet been sought. Perhaps, however, early signs of ER are the basis of acquired resistance to thermal bleaching among corals in Mo’orea^44,53^, as well as only a mild bleaching response by outer reef corals to the El Niño of 2016^54^. Testing for changing frequencies of coral genotypes and phenotypes across an ecological hiatus (e.g., 2010), and evaluating the trends as possible products of ER, is a critical research need for Mo’orea.

### Response diversity

While recent studies have emphasized the importance of taxonomic identity in resolving long-term trends in performance of corals^40^, difficulties in identifying corals have promoted approaches focusing on functional groupings^45,55^. Advances have come from the application of functional groups to coral ecology, but as the present study shows, taxonomic resolution of benthic community structure is required to identify processes mediating changes, for example, response diversity^25^.

Response diversity describes the variation in responses to environmental change of organisms sharing common ecosystem function, and it is thought to play an important role in determining the resilience of ecosystems exposed to anthropogenic disturbances and uncertainty^25,37^. Where taxa differ in their response to an environmental challenge, the emergent property is community resilience (*sensu*^56^) upon exposure to the same disturbance through buffering of the response magnitude. In the present analysis, the destruction and subsequent resilience of the coral community was partitioned into the proportional contribution of the most common coral genera (that together accounted for >64% [mean = 90%] of all corals in every sampling) to reveal taxonomic variation in the rate at which corals were eaten by COTS, and the rate at which they recovered after 2010. In part, these effects reflect feeding preferences of COTS among coral taxa (e.g., *Acropora* > *Pocillopora* > *Porites* > *Montipora* and with *Millepora* rarely eaten in Moorea)^57,58^, differences in the rate at which coral genera grow^59^, and variation in recruitment. Although the analyses of coral recruitment have a coarser taxonomic resolution than coral cover, they suggest that taxonomic variation in rates of recovery begins with recruitment^27^, although differential recruitment is unlikely to drive variation in recovery between depths. While the bio-physical drivers of these effects remain unclear, the products of this response diversity interacted with the feeding preference of COTS to modulate their rates of coral consumption, and the potential to accentuate coral recovery through correspondence of taxa with localized bio-physical conditions promoting their growth. The smaller initial population sizes of the other common taxa (i.e., in 2005), and the weak U-shaped population trajectories (compared to the strong U-shape trajectory for *Pocillopora*) indicate a lower likelihood of ER for these taxa, and thus a reduced probability that they will be well represented on future reefs.

### Summary

Against the backdrop of the coral mortality and profound changes in coral community structure that have occurred since 2015 on the Great Barrier Reef^39,40^ and elsewhere^21,38^, the changes in Mo’orea from 2005–2017 are remarkable. Rather than chronic coral mortality and a ratcheting down of coral reef condition^60^, these reefs have displayed high resilience. These trends are novel, but their importance extends beyond coral cover to suggest that one type of rescue effect (i.e., ER) might be underway, with this effect facilitated by response diversity. The coral community on the outer reef Mo’orea has shown very strong resilience through recovery in response to major disturbances, and this transition has involved the death, and replacement in excess, of 100’s of millions of *Pocillopora* colonies^47^, all of which potentially are genetically unique and reflect the products of an even larger pool of genetically diverse larvae. If this large genetic upheaval within *Pocillopora* populations has facilitated selection to favor genotypes that are more resistant to future conditions, such as warmer seawater, then the recent events in Mo’orea could be an example of ER to promote persistence of pocilloporid corals in the Anthropocene.

## Materials and Methods

Coral community structure was quantified at 10-m and 17-m depth at two sites, 3 km apart on the outer reef of the north shore of Mo’orea (Fig. S1). Sampling for coral community structure began in April 2005 and extended to April 2017, and sampling for coral recruitment began in 2008 (10 m) or 2009 (17 m) and extended at ~6 mo intervals to 2017.

Community structure was evaluated photoquadats (0.5 × 0.5 m) as independent replicates, with these randomly distributed along a single 40-m transect permanently marked at 10-m and 17-m depth at each site. After photoquadrats were randomly located for the initial sampling in 2005, thereafter they were placed annually at the same positions to support a repeated measures design. Photoquadrats were recorded using SLR cameras (Nikon) in housings (Ikelite) that were mounted on a framer. Cameras were attached to strobes (Nikonos SB 105), and images included a scale. Pictures were analyzed by superimposing them with a grid of 200 randomly placed dots, and identifying the taxon beneath each dot. Analyses were completed using CPCe software^61^ until 2016, but thereafter they were analyzed using CoralNET^62^ with manual annotation. The present analysis focused on corals that were scored to genus and included scleractinians and the hydrozoan *Millepora*. Revisions of the taxonomy of fungiids^63^ erected new genera that could not be easily resolved and this taxon was scored by family (Fungiidae).

Coral recruitment was assayed using terra cotta tiles (15 × 15 × 1 cm) that were attached individually using a stainless steel stud. The stud supported the tile horizontally, with the rough surface downward, and a gap of ~1 cm beneath to create a microhabitat favored by coral recruits^64^. 15 tiles were placed at 10-m and 17-m depth at LTER 1 and 2. Tiles were exchanged twice each year, with one deployment in late August/early September, and one in late January/early February; the first measurements of recruitment were obtained in 2008 at 10 m, and in 2009 at 17 m. Prior to deployment, the tiles were seasoned in seawater for ~5 mo, and following deployment, were cleaned in bleach, dried, and scored using a microscope (~40×). Recruits were counted by family^65^. Recruits were integrated over ~6 mo, and annual recruitment was estimated by summing the recruitment averaged by tile within each site and depth for the two samplings y^−1^. To obtain an error estimate on annual recruitment, sites within each depth were treated as replicates to provide a mean ± SE (N = 2 sites).

### Statistical analyses

Coral cover, pooled among taxa and by genus for the four most common corals, were compared among times, depths, and sites with a repeated measures (RM) ANOVA in which time was the RM factor, and sites, and years were fixed effects. Cover was arcsine transformed and assumptions were tested through graphical analysis of residuals. Rates of change in coral cover were calculated for the period of decline caused by COTs (2005–2010, N = 6 y) and ending with Cyclone Oli in February 2010, and also for the period of recovery (2010–2017, N = 8 y). These rates were computed by least squares linear regressions for each quadrat that was sampled repeatedly among years. Slopes of absolute cover against time (% y^−1^) for the decline and recovery phases were compared between sites and depths for the four most common genera using Model I, two way ANOVA. As rates of decline were a function of the initial coral cover, declines also were analyzed on a relative scale obtained by normalizing slopes of cover against time to initial cover (y^−1^). Relative declines were compared between depths and sites with two way ANOVA. To compare rates of absolute and relative decline in coral cover, as well as rates of increase in coral cover, among taxa, depths, and sites, three way PERMANOVA^66^ was used because the statistical design was unbalanced and the data violated the assumptions of ANOVA. Negative data (i.e., declining slopes) were removed by adding a constant dummy values, log transformed, and used to prepare a resemblance matrices by Bray-Curtis dissimilarities. PERMANOVAs were constructed with fixed effects and tested for significance evaluated in a permutational environment (999 permutations) and reported as Pseudo-F values and P_perm_. Coral recruitment was compared between depths for each taxon using t-tests with annualized recruitment averaged between sites as replicates.

Multivariate changes in overall coral community structure were displayed with two-dimensional ordination using non-metric multidimensional scaling (nMDS) in which data (annualized mean percent cover by genus), were square-root transformed data prior to preparing Bray-Curtis dissimilarities from the resemblance matrices. Ordination plots were prepared with 100 restarts until stress stabilized.

Parametric statistics were completed using Systat 13 (Systat Software, Inc., San Jose, CA), and multivariate analyses were completed using PRIMER 6.1 with the PERMANOVA+ add on (PRIMER-E, Quest Research Ltd, Auckland, NZ).

Data for this study are available on the website of the Moorea Coral Reef Long Term Ecological Project: http://mcr.lternet.edu/data and data release 10.6073/pasta/9a32153038bb492da58666e9314bab4d.

## Electronic supplementary material

Supplementary Material
