# Supplementary material for: Implications of high rates of sexual recruitment in driving rapid reef recovery in Mo’orea, French Polynesia
Source: Sci Rep. 2018 Nov 9;8:16615. doi: 10.1038/s41598-018-34686-z (PMC6226471; doi:10.1038/s41598-018-34686-z)

**SUPPLEMENTARY MATERIALS**

**Implications of high rates of sexual recruitment in driving rapid reef recovery in Mo’orea, French Polynesia**

Short title: Death and recovery of a coral reef

Peter J. Edmunds

Fig. S1. Location of Mo’orea in the Society Islands, French Polynesia, with inset showing the location of the study sites (asterisks, LTER1 and 2) on the outer reef of the north shore. Triangle shows location of the Richard B. Gump South Pacific Research Station.


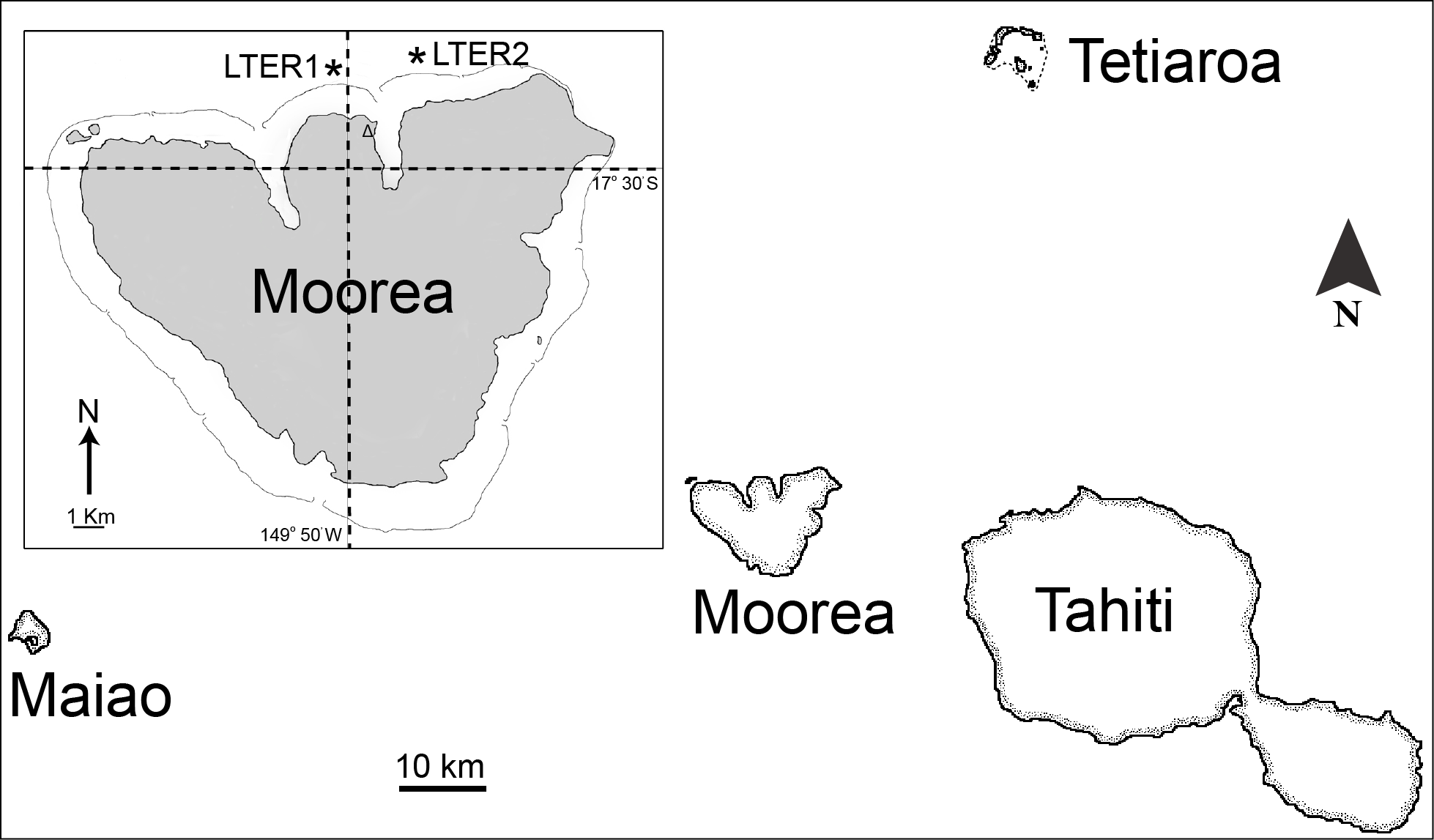

Supplement: Supplementary file 1 — Supplementary Material [file 41598_2018_34686_MOESM1_ESM.docx]
